# Comparison of recovery patterns of gait patterns according to the paralyzed side in Korean stroke patients: Protocol for a retrospective chart review

**DOI:** 10.1097/MD.0000000000012095

**Published:** 2018-08-21

**Authors:** Cheol-Hyun Kim, Hongmin Chu, Chanran Park, Geon-hui Kang, Jihye Seo, Kang-keyng Sung, Sangkwan Lee

**Affiliations:** a Department of Internal Medicine and Neuroscience, College of Korean Medicine, Wonkwang University, Iksan; b Clinical Trial Center, Wonkwang University Gwangju Hospital, Gwangju; c Internal Medicine and Neuroscience, Jangheung Integrative Medical Hospital, Wonkwang University, Jangheung; d Hanbang Cardio-Renal Syndrome Research Center, College of Oriental Medicine, Wonkwang University, Iksan, Jeonbuk, Republic of Korea.

**Keywords:** gait analysis, stroke, Traditional Korean Medicine

## Abstract

**Introduction:**

In Traditional Korean Medicine (TKM), diseases on the left or right side of the human body have been treated differently according to the theory of Donguibogam, which is an encyclopedic source for TKM. In the Wind chapter of Donguibogam, left hemiparesis due to stroke is called Tan, a sort of Hyeol-Byeong, and right hemiparesis due to stroke is called Tan, a sort of Gi-Byeong. As neuroscience develops, it has been shown that the functions of the left and right hemispheres differ, as do the symptoms caused by differently located lesions in the brain. In light of these recent findings and the theory of Donguibogam, it may be useful when treating patients in clinical practice to consider the side of the human body on which symptoms appear. The aim here is to establish whether side-dependent treatments are more effective in treating patients with symptoms on different sides of the body. Specifically, this exploratory study investigates changes in gait pattern among stroke patients with hemiparesis or hemiplegia during gait recovery.

**Methods:**

To develop this protocol, a retrospective review of charts will be used to assess differences in gait recovery patterns among stroke patients with left or right hemiparesis, using gait analysis systems that include GAITRite, G-walk, and Treadmill. The data will be selected from gait analysis performed more than twice in the period from September 1, 2017 to June 31, 2018 at Wonkwang University Gwangju Hospital (WKUGH).

**Results:**

The primary outcomes include spatiotemporal parameters obtained using GAITRite (FAP, velocity, step length, swing time, and stance time); symmetric parameters obtained using G-walk (tilt, obliquity, and pelvis rotation symmetry); and center of pressure (COP) area and velocity as measured by Treadmill.

**Discussion:**

This will be the first study to analyze the gait recovery pattern of stroke patients according to the paralyzed side by comparing spatiotemporal, symmetric, and COP parameters using gait analysis systems. Like all retrospective studies, the present research was subject to certain limitations related to bias (selection bias, recall bias, misclassification bias, confounding value bias), difficulty in assessing temporal relationships, and small sample size. However, these limitations were of less significance here because gait parameters and body side of symptoms of hemiplegia or hemiparesis are relatively clear.

**Conclusion:**

If the use of gait analysis systems (GAITRite, G-walk, and Treadmill) confirms differences in gait recovery pattern among stroke patients according to the paralyzed side, the findings will provide essential evidence for differential treatment of stroke patients on that basis.

## 1 Introduction

Donguibogam the most historic text in Traditional Korean Medicine (TKM), was first published by Dr Heo Jun in 1613 and was included in the United Nations Educational, Scientific, and Cultural Organization cultural heritage list in 2009.^[1]^ According to the Wind chapter of Donguibogam, left or right hemiparesis following stroke is called Tan or Tan, respectively. In addition, left hemiparesis is etiologically classified as Hyeol-Byeong and right hemiparesis of stroke is classified as Gi-Byeong.^[2]^ The treatments differ because the causes differ in terms of body side.

Neuroscience has shown that the brain's left and right hemisphere functions differ, and it follows that symptoms caused by lesions on the 2 sides of the brain are also different. For example, for about 90% of right-handed persons, their language center is in the left hemisphere, and aphasia is mostly caused by left hemisphere lesions.^[3]^ Similarly, hemineglect is associated with functional disorder of the right hemisphere, which appears mostly as left hemineglect.^[4]^ In the same way, recovery pattern also differs according to which side of the brain is damaged. One study reported that left-handed person exhibit a larger transfer effect than right-handed persons in stroke rehabilitation.^[5]^ In light of these recent findings, the idea described in Donguibogam that treatment of stroke patients should be based on hemiparesis side may have great significance for clinical practice.

This study aims to assess the effects of treating left- and right-sided diseases separately for gait rehabilitation of stroke patients based on retrospective chart review. Gait was selected because it is the most important factor in returning stroke patients to daily life.^[6]^ Although some earlier studies^[7,8]^ observed gait recovery progress among stroke patients by comparing their gait with normal subjects, no study has yet observed gait recovery patterns in relation to the paralyzed side. Although Virginia et al reported no difference in prognosis for recovery of motor function in stroke patients with left and right paralysis, their evaluation covered only 3 stages, when patients needed maximal assistance, moderate assistance, or minimal assistance in walking.^[9]^ No study has accurately analyzed stroke patients’ gait using spatiotemporal parameters as proposed here. If we can confirm the existence of differences in gait recovery pattern among stroke patients according to the paralyzed side, this will provide evidence for treating stroke differently on that basis. The intention is to analyze any such differences by comparing the spatiotemporal parameters of left-and right-hemiparesis stroke patients.

## 2 Material and methods

### 2.1 Gait analysis system

#### 2.1.1 The GAITRite system (CIR system Inc)

**Figure 1:**
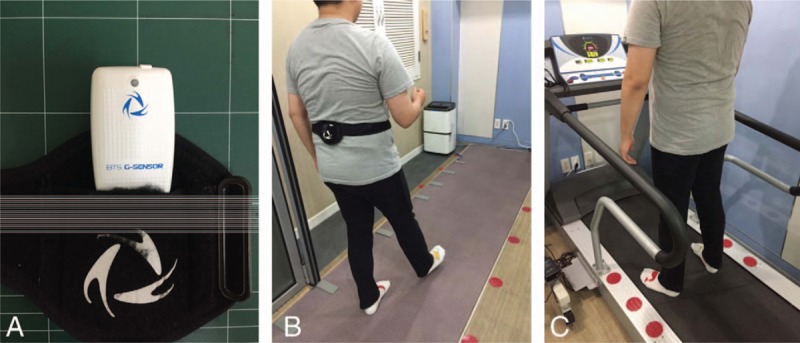
Gait analysis system (A) wireless tri-axial accelerometer of G-walk and Velcro. (B) For gait analysis. The wireless tri-axial accelerometer is attached to the 5th lumbar vertebra of subjects and tightened with Velcro by researchers. Then have a subject walk 6 m GAITRite mat in preferred speed. (C) Next, have a subject stand on Treadmill embedded with force plate for 10 seconds.

When mechanical pressure is applied to the GAITRite mat, embedded sensors are activated (Fig. 1).^[10]^ Following sensor activation, the GAITRite software(version 4.8.5) calculates the elapsed time between heel contact and flat foot stance to obtain temporal and spatial gait parameters such as functional ambulation profile (FAP), velocity, step length, swing time, and stance time. Calculated from walkway data, FAP is a sensitive measure for characterization of dissimilar patients with hemiparesis due to stroke.^[11]^ Walking speed is an important indicator of improved walking ability among stroke patients.^[12]^ Patterson et al have reported that step length, swing time and stance time are also useful gait parameters in this context.^[8]^

#### 2.1.2 The G-walk system (BTS Bioengineering SpA)

G-walk is a gait analysis system that measures the subject's center of mass (COM) by means of a wireless tri-axial accelerometer.^[13]^ The accelerometer is attached to the subjects 5th lumbar vertebra and tightened by means of a Velcro element. The subject then walks to provide parameters including pelvis tilt symmetry, pelvis obliquity symmetry, and pelvis rotation symmetry, as these and other gait symmetry characteristics are increasingly measured and reported in stroke patients.^[8]^ The parameters calculated from the data are transferred wirelessly via Bluetooth for analysis by BTS G-studio software (version 2.8.16.0).

#### 2.1.3 The treadmill analysis system (Zebris Co Ltd FDM-T)

When the subject stands on the Treadmill device, which includes an embedded force plate, foot pressure and center of pressure (COP) are measured and calculated by Zebris FDM software (version 1.10) to obtain parameters such as COP velocity and area.^[14]^ These COP measures are commonly used to assess standing postural control by measuring the extent of body sway.^[15]^

### 2.2 Study design

The study is designed as a retrospective chart review to assess differences in gait recovery pattern between left- and right-hemiparesis stroke patients using the GAITRite, G-walk, and Treadmill gait analysis systems. Subjects will be drawn from stroke patients undergoing gait analysis more than twice between September 1, 2017 and June 30, 2018 at Wonkwang University Korean Medicine Hospitals in Gwangju (WKUGH), those who satisfy the inclusion criteria will be enrolled as participants in the study.

### 2.3 Ethics approval and consent to participate

The retrospective chart review for this protocol (version 1.0) has been granted ethical approval from the institutional review board (IRB) in WKUGH (WKIRB - 18/8, April 28, 2018). This retrospective chart review is being conducted under the supervision of the clinical trial center at WKUGH, monitored by an independent contract research organization. Any change in the protocol will again be submitted for IRB approval prior to implementation. For present purposes, no Data Monitoring Committee (DMC) is needed, and subjects will not be asked to provide written informed consent because retrospective chart review and gait analysis is routine for stroke patients at WKUGH. Subjects may be required to quit the gait analysis following any serious adverse event, and this will be reported to the IRB at WKUGH. In addition, participants may withdraw from gait analysis at any time if they so wish without suffering any disadvantage or constraint. In accordance with the CONSORT 2010 Statement, the results will be published on ClinicalTrials.gov and in specialist journals.

### 2.4 Selection and inclusion criteria

#### 2.4.1 Inclusion criteria

Those who meet all of the following criteria would be selected: WKUGH outpatient or inpatient with hemiparesis due to stroke; stroke patients aged between 19 and 85 years; onset of stroke within the previous 6 months; stroke patients who have undergone gait analysis more twice at WKUGH, with at least 2 weeks interval between first and following gait analysis; right-handed stroke patients; stroke patients who can walk unaided.

#### 2.4.2 Exclusion criteria

Those who have difficulty walking due to other conditions such as musculoskeletal disease.

### 2.5 Gait analysis procedure

**Figure 2:**
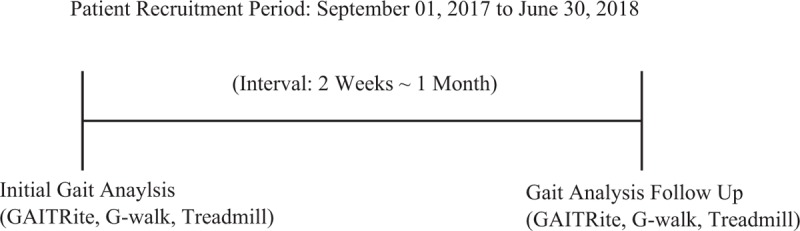
Time schedule of gait analysis gait analysis is a routine examination performed on stroke patients in Wonkwang University Korean Medicine Hospitals in Gwangju (WKUGH). Among the stroke patients who performed the gait analysis more than 2 times during the period from September 1, 2017 to June 30, 2018 in WKUGH, those who satisfies the inclusion criteria becomes the subjects.

The wireless tri-axial accelerometer is attached to the subject's 5th lumbar vertebra and tightened with Velcro by researchers. The subject is then asked to walk 6 m at the preferred speed on a GAITRite mat, and spatiotemporal gait parameters are obtained. The subject then stands on the force plate in the treadmill for 10 seconds to obtain parameters such as COP velocity and area. To ensure measurement quality, the above procedure is repeated twice, and again at least 2 weeks later (Fig. 2).

### 2.6 Outcomes

1. Spatiotemporal parameters obtained by GAITRite: FAP, velocity, step length, swing time, and stance time
2. Symmetric parameters obtained by G-walk: pelvis tilt symmetry, pelvis obliquity symmetry, and pelvis rotation symmetry
3. Parameters while standing obtained by Treadmill: COP area and COP velocity

### 2.7 Statistical analysis

During the initial gait analysis, independent *t* tests or the Mann–Whitney *U* test will be used to verify identity of the 2 groups in terms of Manual Muscle Testing, stroke onset, sex, and age. Paired *t* tests or the Wilcoxon signed test will be used to assess any within-group differences between the first and follow-up gait analysis, and independent *t* tests or the Mann–Whitney *U* test will be used to assess any between-group differences in gait parameters.

### 2.8 Sample size

Because this study is retrospective chart review, all subjects who meet the selection criteria will be included.

## 3 Discussion

Because gait is the most important factor in returning stroke patients to daily life,^[6]^ gait analysis of these patients is conducted regularly in WKUGH. This method is valuable because it provides an objective description of gait quality.^[16]^ Our researchers will conduct the retrospective review to assess whether it is useful to treat left- and right-sided diseases separately as in Donguibogam. For stroke patients who satisfy our selection criteria, statistical analysis will be used to assess difference in gait parameters (spatiotemporal parameters from GAITRite, symmetric parameters from G-walk, and parameters while standing from Treadmill). If the results confirm differences in gait recovery patterns among stroke patients according to the paralyzed side, this will serve as evidence that it is meaningful to treat stroke differently on that basis and to apply this approach to stroke patient gait rehabilitation.

## Author contributions

CK and HC designed and drafted the protocol and manuscript. JS, CP and GK examined the inclusion criteria in clinical practice. KS critically revised the manuscript. SL organized all procedures and revised the protocol. All authors have read and approved the final manuscript.

**Data curation:** Chanran Park, Geon-hui Kang, Jihye Seo.

**Writing – original draft:** Cheol-hyun Kim, Hongmin Chu.

**Writing – review & editing:** Kang-keyng Sung, Sangkwan Lee.
